# 2543 High concentrations of CXCL12 decrease pancreatic adenocarcinoma growth

**DOI:** 10.1017/cts.2018.79

**Published:** 2018-11-21

**Authors:** Emily Vonderhaar, Michael Dwinell, Ishan R. Roy, Donna M. McAllister, Michael B. Dwinell

**Affiliations:** Medical College of Wisconsin

## Abstract

OBJECTIVES/SPECIFIC AIMS: We hypothesized that CXCL12, as a biased dimer variant or secreted at dimer-dominant concentrations, would influence PDAC growth and progression. METHODS/STUDY POPULATION: PDAC cells were genetically manipulated to express dimer-promoting levels of CXCL12. These cells were studied in vitro or orthotopically implanted into the mouse pancreas for in vivo studies. As a second approach, recombinant wild-type or engineered CXCL12 monomer or dimer proteins were applied to cells in culture or administered intra-peritoneal to study the effects on tumor growth. RESULTS/ANTICIPATED RESULTS: Mice engrafted with CXCL12-expressing cells had a better survival rate, delayed tumor growth and smaller tumors. Tumors from these mice had significantly less proliferation, measured by Ki-67 staining. In vitro analysis of CXCL12-expressing cells showed decreased viability and growth rates. Percent of cells in the cell cycle G2 phase was also decreased, suggesting cell cycle progression blockade. Viability of human PDAC cells dose-dependently declined upon wild-type CXCL12 treatment, with the non-motile dimer-dominant dose (1000 nM) exhibiting maximal effect. Treatment in an allogeneic mouse model of PDAC with locked-dimer CXCL12, but not wild-type, reduced tumor burden. DISCUSSION/SIGNIFICANCE OF IMPACT: Our results support the notion that biased CXCL12 signaling may be therapeutically exploited to limit pancreatic cancer progression.

